# Clinical Impact of Hydroxyapatite on the Outcome of Skull Base Reconstruction for Intraoperative High-Flow CSF Leak: A Propensity Score Matching Analysis

**DOI:** 10.3389/fonc.2022.906162

**Published:** 2022-05-04

**Authors:** Shin Heon Lee, Chang-Min Ha, Sang Duk Hong, Jung Won Choi, Ho Jun Seol, Do-Hyun Nam, Jung-Il Lee, Doo-Sik Kong

**Affiliations:** ^1^ Department of Neurosurgery, Chung-Ang University Hospital, Chung-Ang University College of Medicine, Seoul, South Korea; ^2^ Department of Neurosurgery, Samsung Medical Center, Sungkyunkwan University School of Medicine, Seoul, South Korea; ^3^ Department of Otorhinolaryngology-Head and Neck Surgery, Samsung Medical Center, Sungkyunkwan University School of Medicine, Seoul, South Korea

**Keywords:** hydroxyapatite, skull base reconstruction, multilayer technique, cerebrospinal fluid leak, endoscopic endonasal surgery

## Abstract

**Background:**

Despite recent advances in skull base reconstructive techniques, including the multilayer technique during the last decade, complete reconstruction of grade 3 intraoperative high-flow cerebrospinal fluid (CSF) leak remains challenging. This study was designed to investigate the role of injectable hydroxyapatite (HXA) used in the multilayer technique on the clinical outcome of skull base reconstruction for intraoperative high-flow CSF leak.

**Materials and Methods:**

This study enrolled 187 patients who experienced intraoperative high-flow CSF leak after endoscopic endonasal surgery for anterior skull base or suprasellar pathologies between January 2014 and July 2021. All skull base defects were reconstructed using the conventional multilayer technique including a vascularized naso-septal flap (NSF, n = 141) and the combined use of HXA with the conventional multilayer technique (HXA group, n = 46). We retrospectively evaluated the efficacy of the HXA group by 1:2 propensity score matching analysis.

**Results:**

Overall, 17 of 187 patients (9.1%) showed postoperative CSF leaks, resulting in second reconstruction surgery. There were no statistical differences in patient age, sex, body mass index, tumor location, tumor type, and degree of resection, except for the follow-up period between the two groups. The HXA group showed a significantly lower incidence of postoperative CSF leak than the control group (0% vs. 12.1%, p < 0.05). Postoperative lumbar drain (LD) was performed in 8.7% of the HXA group compared to 46.1% of the control group (p < 0.01). CSF leak-related infection rates showed a decreasing tendency in the HXA group compared to the control group (0 vs. 7.1%, p = 0.06). A total of 46 patients in the HXA group were well matched with the control group (92 patients) at a 1:2 ratio. In the propensity score-matched control group, there were higher rates of postoperative CSF leaks than in the HXA group.

**Conclusion:**

The use of HXA combined with the conventional multilayer technique completely reduced postoperative CSF leaks in this study. This technique resulted in reduced CSF leakage, even without postoperative LD, and decreased infection rates. Further randomized comparative studies are required to confirm our findings.

## Introduction

Endoscopic endonasal surgery is now recognized as an essential surgical tool in the skull base field as its indications have expanded into more complex lesions (1–3). Regardless of these advances in endoscopic endonasal surgery, the incidence of postoperative cerebrospinal fluid (CSF) leak is not negligible, varying from 5% to 40% in the literature, which sometimes leads to fatal sequelae such as meningitis or ventriculitis (4–7). In particular, when limited to intraoperative grade 3 high-flow CSF leaks (8), the incidence of postoperative CSF leak was higher, up to 10–15% (8, 9). Therefore, the reconstruction technique for skull base defects is considered the most important issue in endoscopic endonasal surgery.

Since the vascularized naso-septal flap (NSF) method was introduced by Hadad et al., the incidence of CSF leak has been remarkably reduced (10). However, NSF cannot reduce the pulse pressure of the CSF resulting from the abrupt onset of elevated abdominal or intracranial pressure that occurs during the post-anesthetic period. To compensate for this, additional lumbar drainage (LD) for CSF diversion may be required (11, 12). LD for CSF diversion often has significant complications, including headaches related to intracranial hypotension, injection-associated radiculopathy, risk of infection, and restricted mobilization (13, 14). In addition, even the conventional multilayer technique combined with LD still shows approximately 10% of the incidence of postoperative CSF leak when limited to high-flow CSF leaks (13, 15). This implies that a novel technique for safe and effective reconstruction without LD is required to improve the clinical outcomes of endoscopic endonasal surgery.

Hydroxyapatite (HXA) is a suitable material for covering bone defects owing to its biocompatibility and favorable tensile properties (16, 17). Since the injectable form of HXA was introduced, it has been widely used in various fields, including skull base surgery (18, 19). Injectable HXA transforms into a solid form within 5 min and has the advantage of enabling immediate reconstruction by reducing the pulse pressure of the CSF (20). Therefore, it acts as a supplement to the NSF in skull base reconstructions (21). Recent studies on HXA have shown excellent clinical outcomes in terms of the incidence of CSF leak and few side effects (22, 23). To date, little has been reported on the comparative analysis of clinical outcomes between an HXA group and a conventional multilayer group. Here, we evaluated the clinical outcome of a multilayer reconstruction technique using injectable HXA and NSF in patients with intraoperative high-flow CSF leaks by applying a propensity score-matched analysis (1:2) to determine the impact of HXA on the reconstruction outcome.

## Materials and Methods

### Patient Characteristics

The inclusion criteria in this study were patients with anterior skull base or suprasellar non-pituitary tumors who underwent endoscopic endonasal surgery with intra-arachnoidal dissection during tumor removal. These patients received a multilayer reconstructive technique, including an NSF with or without LD for intraoperative high-flow grade 3 CSF leak. The degree of intraoperative CSF leak was assessed using the grading scale suggested by Esposito et al. (8). Between June 2020 and July 2021, 46 consecutive patients with anterior skull base or suprasellar lesions underwent skull base reconstruction using a combined HXA with NSF (HXA group) after endoscopic endonasal surgery. For historical control, 141 consecutive patients with grade 3 CSF leaks who underwent a multilayer reconstruction using NSF were included between January 2014 and May 2020.

Demographic, clinical, and surgical data of the study subjects were collected and reviewed. The degree of each resection was analyzed as “subtotal” (less than 95% of tumor resected), “near-total” (over 95% but less than 100% of tumor resected), and “gross total” (100% of tumor resected) (24). Clinical outcomes including postoperative CSF leak, autologous tissue graft, the presence of LD, and CSF leak-related infection were recorded. Postoperative CSF leak was defined as the presence of persistent rhinorrhea from CSF leak, eventually requiring unplanned second reconstruction surgery. The patients included in the study had a follow-up period of at least 30 days after surgery. All data were anonymized, and the study was approved by the institutional review board of our institution and performed in compliance with the ethical guidelines (approval no. 2021-11-152).

### Surgical Procedure

**Figure 1:**
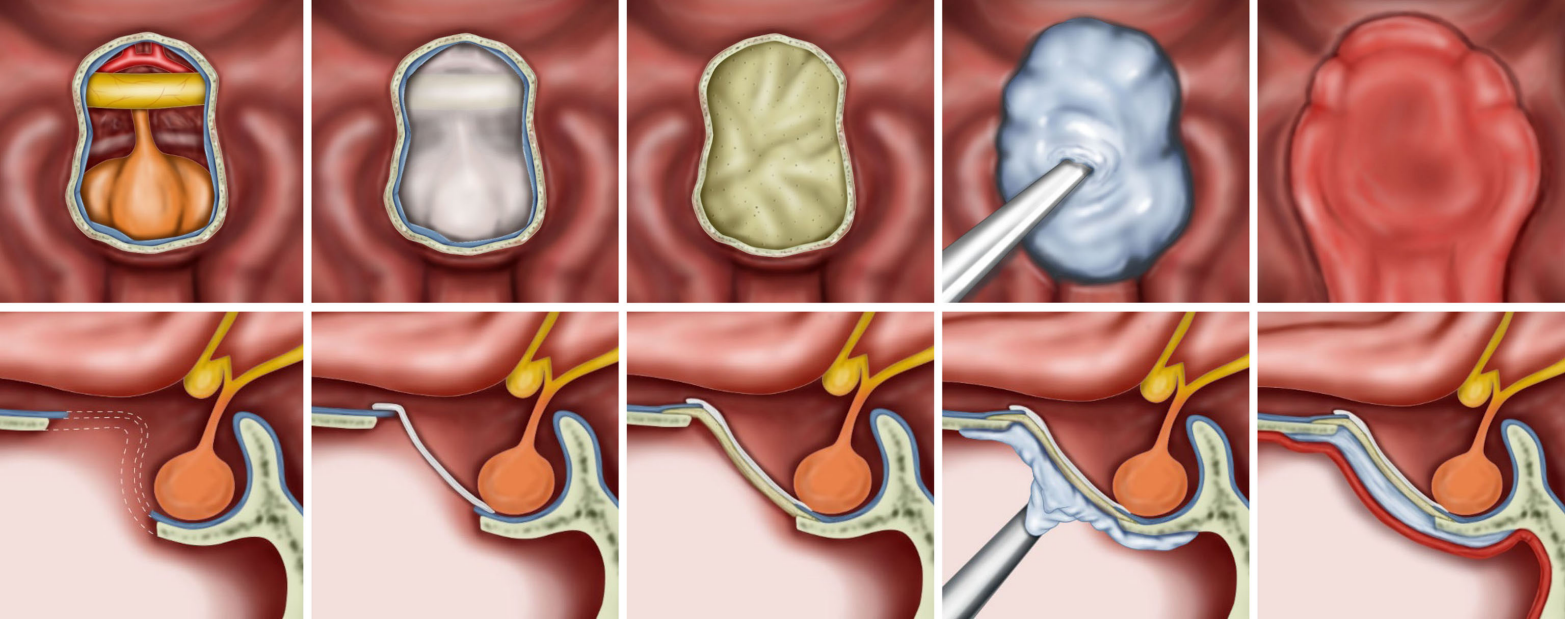
Illustrations for the steps of the multilayer reconstruction technique with hydroxyapatite and a naso-septal flap. The collagen matrix is first placed into the arachnoid defect. Then, an acellular dermal graft is laid over as an on-lay dura graft after being tailored according to the size of the bone defect. Injectable hydroxyapatite is applied as an additional closure of the sellar defect. Finally, a naso-septal flap covers the above materials.

**Figure 2:**
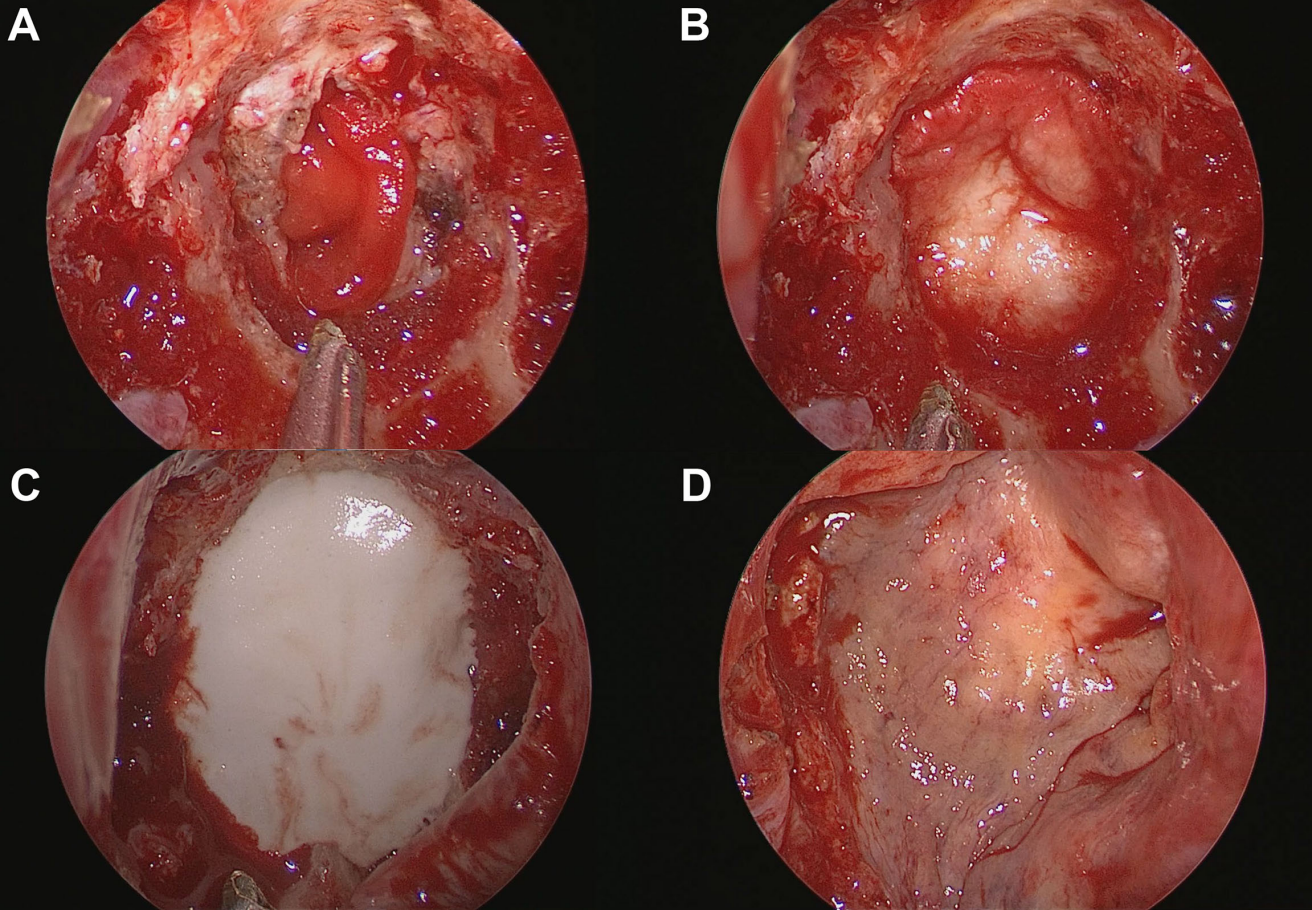
Process of skull base reconstruction using a multilayer technique with hydroxyapatite and a naso-septal flap. **(A)** Collagen matrix placed into the arachnoid defect. **(B)** Acellular dermal graft laid over as an on-lay dura graft after being tailored according to the size of the bone defect. **(C)** Injectable hydroxyapatite was applied as an additional closure of the sellar defect. **(D)** A naso-septal flap covers the above materials.

Details of endoscopic endonasal surgery have been described previously (25, 26). All surgeries were performed by an endoscopic skull base surgery team comprising a neurosurgeon (D.K.) and an otorhinolaryngologist (S.D.H.) at a single institution. Meticulous hemostasis was achieved after tumor resection. We adapted multiple-layer watertight techniques to reconstruct high-grade CSF leakage (grade 3) in patients, where all boundaries of the surgical window were surrounded by rigid skull base bone with reliable hardness and sufficient width. No attempt has been made to re-approximate the dura primarily or secondarily. When a large defect of the arachnoid membrane remained in the area where the tumor was removed, the defect was closed with a collagen matrix (Duragen^®^, Integra LifeSciences, New Jersey, USA). Then, an acellular dermal graft with a thickness of 1 mm (AlloDerm^®^, BioHorizons, Alabama, USA or MegaDerm^®^, L&C BIO, Gyeonggi-do, Korea) was laid over as an on-lay dura graft after being tailored according to the size of the sellar bone defect. The mucosal membrane surrounding the bony defect was completely removed, and the bony surface was in contact with HXA and NSF as much as possible. Before injecting the HXA, it was important to maintain the dry condition of the bony surface. Injectable HXA (Hydroset^®^, Stryker Leibinger, Freiburg, Germany) was applied over the surrounding bony area to fully cover the bone defect. At this step, we identified no intraoperative CSF leak from the reconstructed graft site 5–10 minutes after injection. An NSF was covered on the above grafts, and as a result, the HXA was not exposed to the nasal cavity (**Figures 1**, **2**). Finally, pieces of nasal packing material (Merocel^®^, Medtronic, Minneapolis, USA) were inserted into the nasal cavity to compress and stabilize the graft. The key points of the reconstruction procedure are demonstrated in **Supplementary Video 1**.

In contrast, for the control group, the buttonhole method using either a fascia lata graft or allodermis, consisting of inlay and on-lay double layers, was used after plugging a collagen matrix into the arachnoid defect. In some cases, a septal bone graft was placed along with the gasket technique for bony defects. Then, the defect site was fully covered using a pre-prepared NSF that was already harvested at the beginning of the operation; it was also verified that the margin of the NSF was completely grafted on the denuded skull base. Pieces of nasal packing material were also inserted to compress and stabilize the graft.

### Postoperative Evaluation and Management

**Figure 3:**
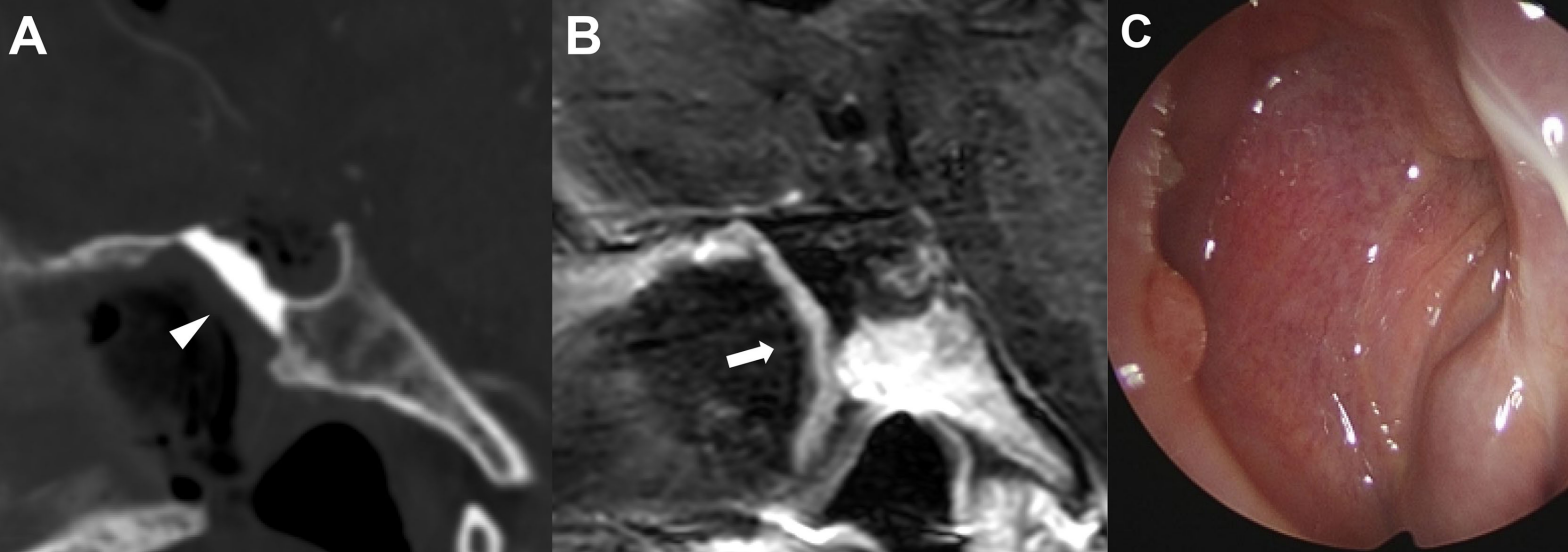
Representative images of skull base reconstruction in endoscopic endonasal surgery of craniopharyngioma. **(A)** Postoperative sagittal computed tomography image of the operative area with hydroxyapatite *in situ* (arrowhead). **(B)** Postoperative sagittal magnetic resonance imaging with contrast enhancement indicating the integrity of vascularized naso-septal flap (arrow). **(C)** Complete sealing status of hydroxyapatite by the flap 3 months after surgery.

After surgery, three antibiotics (third-generation cephalosporin, quinolone, and metronidazole) were administered intravenously for 3-5 days in all patients. Postoperative follow-up examinations were performed in all patients to monitor for CSF leaks. Rhinological examination was performed on the second and fifth postoperative days. The packing materials, such as Merocel in the nasal cavity, were removed, and then the reconstructed skull base was evaluated with an endoscope to verify the viability of the NSF and to check for CSF leaks. Postoperative imaging was routinely performed within 1 day after surgery to identify the degree of the tumor resection and the integrity of the NSF (**Figures 3A, B**).

After discharge, patients were followed up in neurosurgery, otorhinolaryngology, and endocrinology departments in outpatient clinic settings. Pituitary function was evaluated one month after surgery to determine whether hormone replacement therapy was needed. Follow-up magnetic resonance imaging was routinely performed 6 months postoperatively in each patient. Within 2 months after surgery, the nasal cavity was cleaned and examined monthly to monitor the nasal mucosa status until healing was complete (**Figure 3C**).

### Statistical Analysis

R version 4.0.4 (R Foundation for Statistical Computing, Vienna, Austria) was used for all statistical analyses. For categorical variables, data were expressed as frequencies and percentages. The chi-square test and Fisher’s exact test were used to analyze categorical variables using contingency tables. For continuous variables, data were expressed as the mean ± standard deviation. The mean differences between the HXA and control groups were analyzed using Student’s *t*-test. Statistical significance was set at *p* < 0.05.

Propensity score matching was undertaken to compensate for group-wise imbalances in baseline characteristics that had the potential to skew the outcomes. Score matching of each patient, which reflected each patient’s age, sex, body mass index (BMI), lesion location, and degree of resection, was conducted using the 1:2 optimal matching method in the Matchit package of R version 4.0.4. The caliper width was set to 0.1 times the standard deviation of the logit of the propensity score to prevent poor matches. The quality of each match was assessed by comparing selected variables in propensity score-matched patients using the standardized mean difference, in which an absolute standardized difference of greater than 10% is suggested to represent a meaningful covariate imbalance.

## Results

### Baseline Characteristics

A total of 187 patients that received a multilayer reconstruction technique following an intraoperative grade 3 CSF leak were included in the study. There were 101 women and 86 men with a mean age of 46.2 years. A total of 141 patients (75.4%, control group) underwent a multilayer reconstruction using NSF with or without LD, and 46 patients underwent a multilayer reconstruction with both NSF and HXA (24.6%, HXA group). The mean BMI was 24.8 ± 4.5 kg/m^2^. Craniopharyngioma was the most common pathology (129 cases), followed by meningioma (49 cases), germinoma (3 cases), epidermoid cyst (2 cases), dermoid cyst (1 case), teratoma (1 case), neurofibroma (1 case), and pleomorphic xanthoastrocytoma (1 case). Regarding the surgical location, 46 lesions (24.6%) were at the anterior skull base, and 141 lesions (75.4%) were in the suprasellar region. Ten patients (5.3%) had a history of endoscopic endonasal surgery. Ninety-six (51.3%) patients underwent gross total resection, 54 (28.9%) had near-total resection, and 37 (19.8%) had subtotal resection of the target lesion. The mean follow-up period was 34.5 ± 26.8 months (**Table 1**).

**Table 1:** Demographic, disease, and surgical characteristics of patients.

	Total			Propensity score matched		
Variables	HXA group (n=46)	Control group (n=141)	*P*-value	HXA group (n=46)	Control group (n=92)	*P*-value
Female	25 (54.4%)	76 (54.3%)	1	25 (54.4%)	53 (57.6%)	0.720
Age (years)	50.5 ± 17.0	44.8 ± 18.1	0.061	50.5 ± 17.0	49.0 ± 16.9	0.630
Body mass index (kg/m^2^)	25.3 ± 4.1	24.6 ± 4.6	0.366	25.3 ± 4.1	25.2 ± 4.8	0.870
Pathology					
Craniopharyngioma	28 (60.9%)	101 (71.6%)		28 (60.9%)	67 (72.8%)	
Meningioma	13 (28.3%)	36 (25.5%)		13 (28.3%)	23 (25.0%)	
Germinoma	3 (6.5%)	0		3 (6.5%)	0	
Epidermoid cyst	0	2 (1.4%)		0	2 (2.2%)	
Dermoid cyst	0	1 (0.7%)		0	0	
Teratoma	0	1 (0.7%)		0	0	
Neurofibroma	1 (2.2%)	0		1 (2.2%)	0	
Pleomorphic xanthoastrocytoma	1 (2.2%)	0		1 (2.2%)	0	
Location			0.844			1
Anterior skull base	12 (26.1%)	34 (24.1%)		12 (26.1%)	23 (25.0%)	
Suprasellar	34 (73.9%)	107 (75.9%)		34 (73.9%)	69 (75.0%)	
Prior endoscopic skull base surgery	4 (8.7%)	6 (4.3%)	0.265	4 (8.7%)	4 (4.4%)	0.441
Degree of resection			0.623			0.857
Gross-total resection	24 (52.2%)	72 (51.1%)		24 (52.2%)	50 (54.3%)	
Near-total resection	15 (32.6%)	39 (27.7%)		15 (32.6%)	26 (28.3%)	
Subtotal resection	7 (15.2%)	30 (21.3%)		7 (15.2%)	16 (17.4%)	
Mean follow-up period (months)	7.5 ± 4.1	43.4 ± 25.0	**<0.001**	7.5 ± 4.1	41.2 ± 24.0	**<0.001**

HXA, hydroxyapatite. Bold values denote statistical significance at the p < 0.05 level.

### Reconstruction Outcomes and Related Complications

Postoperative CSF leak occurred in 17 of 187 patients (9.1%). In the 17 patients with CSF leak, dehiscence of non-vascularized constructs such as fascia graft (8 patients, 4.3%) and detachment or displacement of NSF (8 patients, 4.3%) were the main reasons for failure. The median time interval to CSF leak was 10 days (range, 5–23 days). Autologous tissue grafts were performed in 119 of 187 patients (63.6%). Postoperative LD was prophylactically performed in 69 patients (36.9%). CSF leak-related infection occurred in 10 patients (5.3%). Bacterial meningitis was confirmed in six patients (3.2%), fungal meningitis in two patients (1.1%), and ventriculitis in two patients (1.1%). One case (0.5%) was a mortality case in which a patient with bacterial meningitis, followed by fungal meningitis, progressed to a septic condition (**Table 2**).

**Table 2:** Clinical outcome comparison between the HXA group and the control group.

	Total			Propensity score matched		
Outcomes	HXA group (n=46)	Control group (n=141)	*P*-value	HXA group (n=46)	Control group (n=92)	*P*-value
CSF leak	0	17 (12.1%)	**0.014**	0	11 (12.0%)	**0.015**
Dehiscence of non-vascularized constructs		8 (5.7%)			6 (6.5%)	
Displacement of NSF		8 (5.7%)			7 (7.6%)	
No definite leakage point identified		3 (2.1%)			1 (1.1%)	
Time interval to CSF leak (days)		10 (5–23)			10 (5–23)	
Autologous tissue graft	0	119 (84.4%)	**<0.001**	0	79 (85.9%)	**<0.001**
Postoperative LD	4 (8.7%)	65 (46.1%)	**<0.001**	4 (8.7%)	38 (41.3%)	**<0.001**
CSF leak/LD		7/65 (10.8%)			2/38 (5.3%)	
CSF leak/No LD	0/42 (0%)	10/76 (13.2%)	**0.014**	0/42 (0%)	9/54 (16.7%)	**0.004**
CSF leak-related infection	0	10 (7.1%)	0.063	0	4 (4.4%)	0.151
Bacterial meningitis		6 (4.3%)			1 (1.1%)	
Fungal meningitis		2 (1.4%)			1 (1.1%)	
Ventriculitis		2 (1.4%)			2 (2.2%)	

HXA, hydroxyapatite; NSF, naso-septal flap; CSF, cerebrospinal fluid; LD, lumbar drainage. Bold values denote statistical significance at the p < 0.05 level.

### Propensity Score-Matched Analysis Between the Subgroups

**Figure 4:**
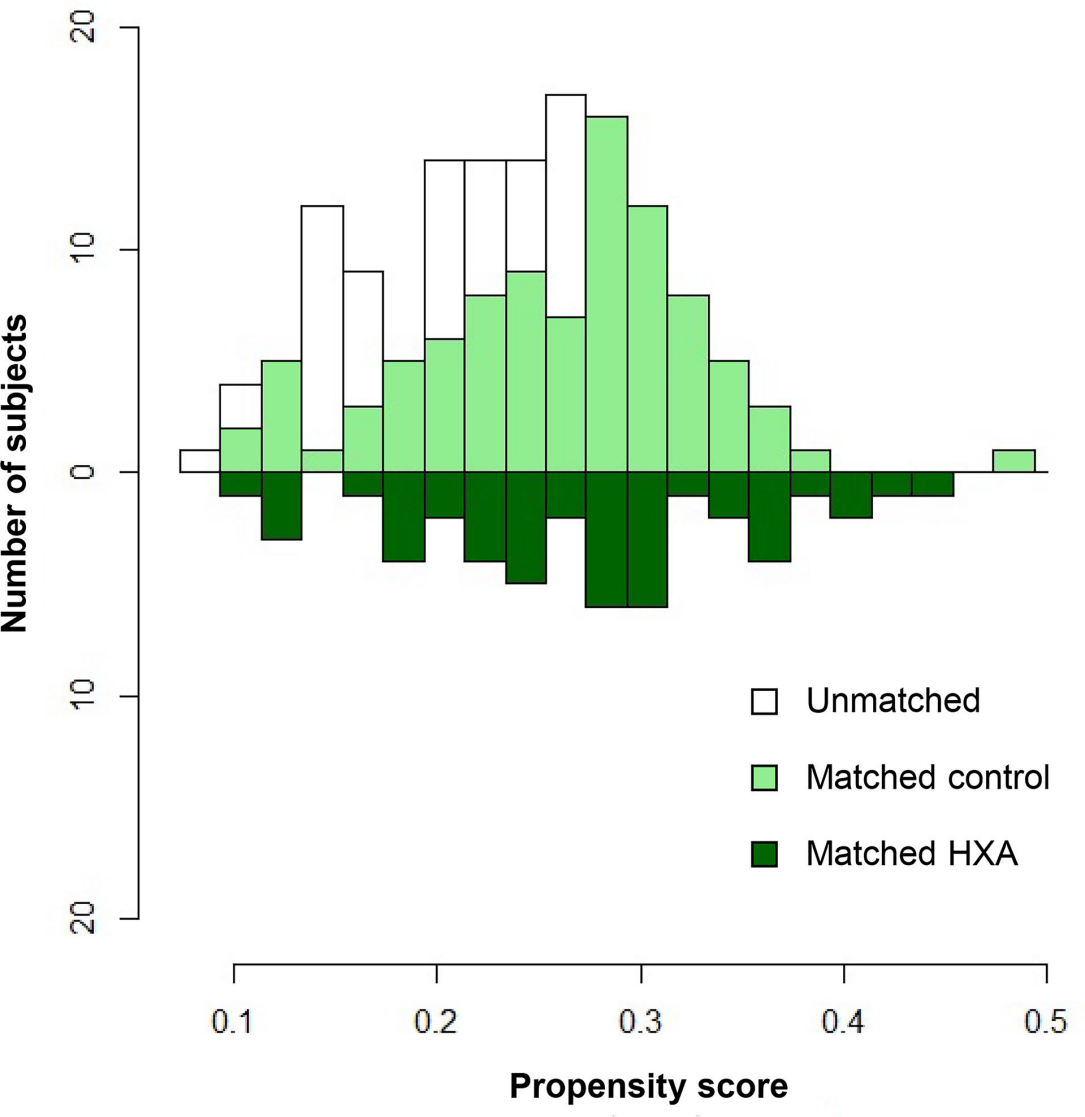
Mirrored histogram of propensity scores for patients who underwent skull base reconstruction with the conventional multilayer technique and combined use of hydroxyapatite with the multilayer technique. Colored areas represent propensity-matched cohorts.

Propensity score matching (1:2) created a total of 46 matching sets (46 and 92 patients in the HXA and matched control groups, respectively). After matching, covariates were well balanced between the two groups, with a standardized mean difference of less than 10% for all selected variables (**Supplementary Figure 1**). The distributions of the propensity scores between the groups are shown in **Figure 4**. The group characteristics were comparable in the matched subjects, but the mean follow-up period remained significantly higher in the control group (41.2 ± 24.0 months) than in the HXA group (7.5 ± 4.1 months) due to the modification of the surgical method in this period (*p <* 0.001) (**Table 1**). The reconstruction outcome analysis after propensity score matching is shown in **Table 2**. The control group exhibited more postoperative CSF leaks (12.0 vs. 0%, *p* = 0.015) and increased need for autologous tissue grafts (85.9 vs. 0%, *p <* 0.001). Additionally, in contrast to the HXA group, wherein there was no CSF leakage (even among the patients for whom postoperative LD was not performed), there was CSF leakage in 16.7% of patients in the control group without postoperative LD (*p* = 0.004). The CSF leak-related infection rate was lower in the HXA group than in the control group (0 vs. 4.4%); however, statistical significance was not observed (*p* = 0.151).

## Discussion

To prevent CSF leak after surgery, several methods that combine autologous, allogeneic, and artificial materials have been implemented for skull base reconstructions (27–29). The traditional reconstruction methods for CSF leak include the use of an abdominal fat graft and insertion of a postoperative LD for the CSF (30, 31). However, abdominal fat grafting requires an additional abdominal incision and can lead to harvest site morbidity (32). The development of a vascularized NSF method has dramatically reduced CSF leakage compared to the non-vascularized reconstruction method using an abdominal fat graft or fascia lata graft (10, 33, 34). However, there is a caveat that sufficient experience and skillful dexterity must be accumulated to properly harvest and apply the flap (6). In addition, it takes time for the NSF to completely adhere to the skull base and the normal mucosa at the surgical site (4, 10). If the CSF flow is not effectively blocked or bypassed before the flap is settled, CSF leaks may occur despite the use of the NSF (4, 10). Therefore, CSF diversion through LD for a certain period after surgery is frequently needed (13).

However, LDs have several disadvantages that must be balanced against their advantages. The recommended duration of LD maintenance varies between studies, and no consensus has been reached (8, 13). In our series of patients, postoperative LD was maintained for at least 3 to 5 days. Maintaining LDs is not only time-consuming but also carries a risk of complications. While the drainage tube is inserted, the patient’s ambulation becomes very uncomfortable, and in most cases, the patient has to rest in bed. Although it is not very common, prolonged bed rest increases the probability of serious side effects such as pneumonia, pulmonary embolism, and deep vein thrombosis due to immobilization (35). Moreover, CSF overdrainage increases the possibility of complications such as postural headache, subdural hemorrhage, and brain herniation (36, 37). Meningitis and other infections have been reported in 4–10% of LD cases (38). The longer the duration of LD, the higher the infection rate (39). Therefore, it is important to find a novel skull base reconstruction method to minimize the insertion of LD and to reduce the duration of drainage, even when drainage is required.

HXA is an artificial bone material that has been previously used to reconstruct bone defects (18, 19). Injectable HXA (Hydroset^®^) can be applied using a syringe and can be used in the skull base (20, 21). HXA hardens within a few minutes, but it can be made into the desired shape while it is in its semi-solid state before hardening (20, 21).

Although HXA is currently widely used for bone reconstruction, there are concerns about its toxicity, immunogenicity, difficulty in reoperation, and high cost (24). Several animal studies have confirmed that HXA induces minimal inflammation *in vivo* and has favorable biocompatibility for bone reconstruction (40–42). Nevertheless, it can cause some problems, including recurrent crust formation, particularly if the substance is exposed to the nasal cavity (43). Importantly, since it is an artificial material, the possibility of infection must be considered due to its nature as a foreign body (43, 44). Therefore, injectable HXA should be carefully managed within the nasal cavity. It is important to fully cover the injected HXA with an NSF as an on-lay. As making an NSF requires experience, management of HXA requires careful attention and experience. On the other hand, there are clear advantages to the use of HXA, including reductions in harvest site morbidity due to autologous tissue grafting and reduced LD insertion.

Our findings showed acceptable clinical outcomes in terms of HXA-associated complications. Kim et al. previously reported on a multi-layer on-lay reconstruction technique using a fibrin sealant patch (TachoSil^®^, Takeda, Osaka, Japan), HXA, and an NSF. They reported postoperative meningitis without cultured microorganisms in 13.5% of patients who underwent reconstruction (22). However, no such cases were identified in our study, and there was no HXA-associated infection or crust formation during the follow-up period, which has been reported in previous studies (22, 43, 45). This could be attributed to isolation from the intradural space using multilayers, such as collagen matrices and acellular dermal grafts, as well as due to separation from the intranasal cavity with the aid of the NSF. On-lay sealing using a fibrin sealant patch alone is relatively thin, and this material is known to biodegrade within a period of about 12 weeks (46). On the other hand, the inlay and on-lay dual configuration of the collagen matrix and acellular dermal graft can seal with more stability. Each implant can function as a scaffold for the reconstitution of host collagen, promoting fibroblast ingrowth and neovascularization until its resorption after 6 months to 1 year (47, 48). Therefore, the possibility of direct contact between HXA and the intra-dural space can be reduced, resulting in a low incidence of postoperative meningitis.

The primary objective of this study was to evaluate the reconstruction outcomes of the multilayer technique with HXA and NSF for high-flow CSF leaks. Reducing selection bias is critical for accurately assessing repair outcomes. Previous reports have covered all types of pathologies with different classes of CSF leaks. However, our study deliberately focused on the highest-grade CSF leak (grade 3) to confirm the effectiveness of our reconstruction method. We defined a “high-flow CSF leak” as a situation requiring intra-arachnoidal dissection and violation of a ventricle or cistern, which requires a more robust skull base reconstruction. As this was an inclusion criterion, extra-arachnoid lesions, such as pituitary adenoma or Rathke’s cleft cyst, were excluded from patient selection. In addition, skull base defects after operations on posterior fossa tumors may not be entirely covered by the HXA and NSF due to the extent of this type of large bone defect. Therefore, additional reconstruction and CSF diversion are required in these cases, and inconsistent reconstruction methods in a case-by-case manner may affect the accurate comparison of CSF leak results in this study. Thus, non-pituitary tumors of the skull base region requiring intra-arachnoid dissection, except for lesions in the posterior fossa, were selected as the inclusion criteria for our study.

Reconstruction after endoscopic endonasal surgery remains a long-standing challenge. Our multilayer technique using HXA and NSF effectively prevented CSF leakage and associated fatal infections. Although the decision for or against LD placement is made by the surgeons, it is worth noting that there was no CSF leakage in 42 of the 46 patients in the HXA group who did not undergo postoperative LD. Compared with the conventional method, our method prevented CSF leakage while reducing the frequency of postoperative LD. In addition, it has the advantage of reducing harvest site morbidity by decreasing the need for autologous tissue grafts. HXA, which can harden immediately within minutes, can advantageously create a barrier that can be relied upon when non-vascularized constructs such as fascia lata or NSF are being organized with surrounding tissues. Possibly due to the inlay and on-lay coverage of HXA, there were no HXA-associated complications, such as delayed infection, graft protrusion, fistula formation, or crust formation (22, 43, 45).

This study has some limitations. This study was designed as a retrospective study rather than a prospective randomized method, which may have led to bias due to the inherent nature of the study design. Also, data were collected from a single institution by a single surgical team. There was a difference in the follow-up period between the control group and the HXA group due to the modifications in the reconstruction method during the investigated period. The mean follow-up period of 7.5 ± 4.1 months in the HXA group might not be sufficient to detect delayed complications. In addition, analysis of skull base defect size could not be performed because of the retrospective nature of the study. Since this factor may be closely related to the CSF leakage rate, further analysis is required. The use of HXA may make revision surgery challenging; however, this aspect could not be analyzed in this study. Multicenter studies with a prospective randomized design and those that include larger patient cohorts and longer follow-up durations will help to verify the clinical utility of the reconstruction method presented here.

## Conclusion

The multilayer technique using HXA and NSF provided a reliable reconstructive outcome for high-flow CSF leaks after endoscopic endonasal surgery. This technique effectively prevented CSF leakage while reducing the demand for postoperative LDs and lowering the infection rate.

## Data Availability Statement

The original contributions presented in the study are included in the article/**Supplementary Material**. Further inquiries can be directed to the corresponding author.

## Ethics Statement

The studies involving human participants were reviewed and approved by the institutional review board of Samsung Medical Center. Written informed consent for participation was not required for this study in accordance with the national legislation and the institutional requirements.

## Author Contributions

SHL: Methodology, Data curation, Resources, Validation, Investigation, Formal analysis, Visualization, Writing - original draft. CMH: Data curation, Resources. SDH: Data curation, Resources, Validation. JWC: Supervision. HJS: Supervision. DHN: Supervision. JIL: Supervision. DSK: Conceptualization, Methodology, Data curation, Resources, Validation, Project administration, Writing - review & editing. All authors contributed to the article and approved the submitted version.

## Conflict of Interest

The authors declare that the research was conducted in the absence of any commercial or financial relationships that could be construed as a potential conflict of interest.

## Publisher’s Note

All claims expressed in this article are solely those of the authors and do not necessarily represent those of their affiliated organizations, or those of the publisher, the editors and the reviewers. Any product that may be evaluated in this article, or claim that may be made by its manufacturer, is not guaranteed or endorsed by the publisher.
