# Supplementary material for: Clinical Impact of Hydroxyapatite on the Outcome of Skull Base Reconstruction for Intraoperative High-Flow CSF Leak: A Propensity Score Matching Analysis
Source: Front Oncol. 2022 May 4;12:906162. doi: 10.3389/fonc.2022.906162 (PMC9116718; doi:10.3389/fonc.2022.906162)
Supplement: Supplementary file 1 [file DataSheet_1.docx]

Supplementary Material


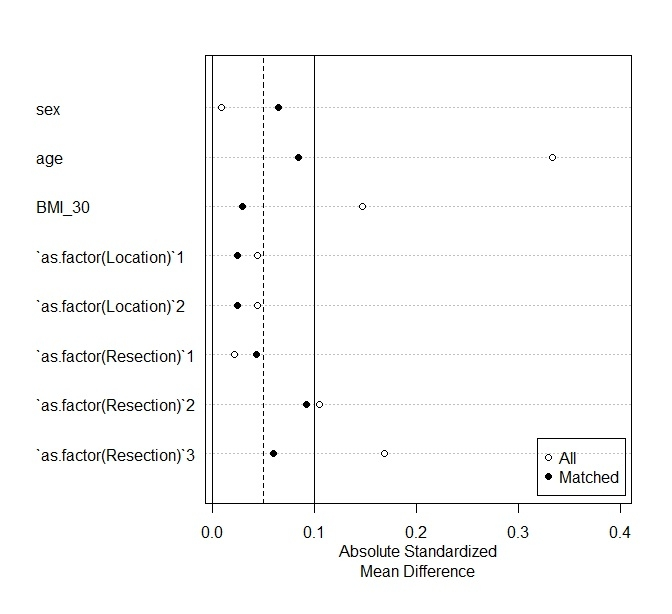


**Supplementary Figure 1.** Standardized mean differences plot before and after matching. All differences for the matched observations are within the balance limits of 0.1. BMI, body mass index.
